# Haptoglobin Genotype Is a Determinant of Hemoglobin Adducts and Vitamin E Content in HDL

**DOI:** 10.1155/2018/6125420

**Published:** 2018-05-20

**Authors:** Hagit Goldenstein, Nina S. Levy, John Ward, Tina Costacou, Andrew P. Levy

**Affiliations:** ^1^Technion Faculty of Medicine, Technion Israel Institute of Technology, 1 Efron Street, Haifa, Israel; ^2^Department of Epidemiology, University of Pittsburgh, 3512 Fifth Avenue, Pittsburgh, PA 15213, USA

## Abstract

Haptoglobin (Hp) is an abundant hemoglobin- (Hb-) binding serum protein and a constituent of the HDL proteome. In man, there exists a common polymorphism at the Hp locus with two common alleles defined by the presence (Hp 2 allele) or absence (Hp 1 allele) of a 1.7 kb in-frame partial duplication of exons 3 and 4 of the Hp gene. Numerous studies have demonstrated that the Hp 2-2 genotype is associated with a 3–5-fold increase in vascular disease among individuals with diabetes mellitus (DM). Increased Hp-Hb complex has been shown to be associated with the HDL of Hp 2-2 DM individuals. Hb-associated HDL has been proposed to result in the oxidation of HDL and the consumption of antioxidants in HDL, such as vitamin E, rendering the HDL further susceptible to oxidation. In this study, we set out to identify proteins which become cross-linked to Hb in HDL and to measure vitamin E in HDL as a function of the Hp genotype. We report on the identification of a novel 72 kd Hb reactive species which is cross-linked to HDL and demonstrate that vitamin E in HDL is decreased in Hp 2-2 DM individuals.

## 1. Introduction

Haptoglobin (Hp) is a plasma protein which binds free hemoglobin (Hb) and prevents oxidative damage between the heme iron of Hb and proteins and lipids [1]. The ability of Hp to neutralize potential oxidation from the heme in Hb is especially important in DM, where there is increased free extracorpuscular Hb and increased systemic oxidative stress due to hyperglycemia [2]. In man, there exists a common polymorphism at the Hp locus with two common alleles defined by the presence (Hp 2 allele) or absence (Hp 1 allele) of a 1.7 kb in-frame duplication of exons 3 and 4 of the Hp gene. Multiple studies have shown that the Hp 2-2 genotype, representing 40% of the total population, confers a higher risk of developing vascular complications in the setting of DM than other Hp genotypes. For example, the Israel Cardiovascular Vitamin E study (ICARE) [3] and the Strong Heart Study [4] demonstrated that Hp 2-2 DM individuals were three to five times more likely to suffer from cardiovascular disease (CVD) than DM individuals with the Hp 1-1 or 2-1 genotypes.

The mechanism of increased vascular risk in Hp 2-2 individuals is likely due to the impaired ability of the Hp 2-2 protein to prevent Hb-driven oxidation as compared to the Hp 1-1 protein. This mechanism is supported by results from randomized placebo-controlled clinical trials that have demonstrated that vitamin E is effective in reducing vascular disease risk of DM Hp 2-2 individuals [5, 6].

High-density lipoprotein (HDL) has been proposed to play a major role in preventing cardiovascular complications by mediating the removal of cholesterol from the macrophage and the vessel wall in a process referred to as reverse cholesterol transport (RCT). However, clinical trials aimed at increasing HDL levels have been largely ineffective in decreasing cardiac events [7]. This has led to suggestions that HDL therapy should be targeted at improving the quality of HDL rather than its quantity [8]. Hp is known to bind to HDL on the ApoA1 protein. Studies have shown that more Hb is associated with HDL in Hp 2-2 DM compared to Hp 1-1 DM [5] and that RCT is impaired in Hp 2-2 DM individuals [9]. Furthermore, vitamin E supplementation has been shown to improve HDL-stimulated cholesterol efflux and reduced HDL lipid peroxidation in Hp 2-2 DM individuals [5]. In this study we sought to understand and identify how Hb complexed to HDL might result in oxidative modification of HDL and how this might affect HDL functionality.

## 2. Methods

### 2.1. Hb Preparation

Blood was drawn from healthy adults. The blood was centrifuged for 10 min at 188 ×g, and the red blood cells (RBCs) were separated from the plasma. RBCs were washed three times by adding phosphate-buffered saline and centrifuging for 10 min at 188 ×g and lysed with water using ten times the volume of blood. After another centrifugation at 15,000 ×g for 45 min, the hemolysate was separated into aliquots and stored at −80°C until use. Before each experiment, catalase and superoxide dismutase were removed from the Hb preparation by DEAE-Sepharose anion-exchange chromatography [10].

### 2.2. Hp Preparation

Hp from plasma donated by 4 healthy individuals (2 samples for each Hp 1-1 and Hp 2-2) was affinity purified using a goat anti-Hp antibody column. Hp was stored at −80°C until use [11].

### 2.3. HDL Purification

HDL was purified by two different methods: first, by using macroultracentrifugation as was described by Asleh et al. [5] and second, by using density gradient microultracentrifugation. Briefly, plasma (500 *μ*l) was incubated with 20 *μ*l of a heparin solution (5000 U/ml) and 50 *μ*l of 1 M MnCl_2_ at 4°C. After 30 min of incubation, the samples were centrifuged for 20 min at 1500 ×g and 4°C. The upper layer was transferred to a microultracentrifuge tube and 300 *μ*l of 1.95 M NaCl and 76.5 M of NaBr solution (1.478 g/cm^3^) were added to it. The tubes were centrifuged for 2.5 hours at 16°C and 120,000 ×g. After the centrifugation, 300 *μ*l from the upper layer were removed and dialyzed overnight against a 1.006 g/cm^3^ 0.186 M NaCl solution with 1.26 mM EDTA. The samples were stored at 4°C.

### 2.4. HDL and Hb-Hp Complex Oxidation

Eight different Hb-Hp complexes were formed by incubating Hb and Hp (1 : 1 molar ratio) for 15 min at 25°C and then incubating with HDL for one hour. H_2_O_2_ (1 : 5 molar excess) was added to Hb-Hp complexes for 30 min at 37°C and the samples were passed through a PD-10 column, in order to remove H_2_O_2_. The samples were diluted to a final concentration of 500 nM and were loaded on SDS-PAGE gels and transferred onto a PVDF membrane. In order to prevent nonspecific antigen binding, the membrane was incubated with 1% milk for 30 min in 25°C. A rabbit anti-Hb antibody (DAKO, dilution 1 : 2000) was added overnight: incubation was performed at 4°C. After washing the membrane for 10 min × 3 times with TBST, polyclonal goat anti-rabbit HRP-conjugated antibody (DAKO, dilution 1 : 2000) was added for 30 min at 25°C. Proteins were identified and quantified using an ECL substrate. The membrane was washed with a reblot buffer purchased from Mercury Scientific & Industrial Products Ltd. (Rosh Ha-ayin, Israel), and the procedure was repeated with rabbit anti-Hp antibody. The potential role of Hb-induced tyrosine radicals in generating Hb-HDL cross-links was tested by studying the effect of different concentrations (10–400 nM) of hydroxyurea on HDL Hb cross-linking.

### 2.5. Sequencing of the Hb Immunoreactive 72 kd Band in HDL

Two samples of 72 kd bands prepared by two different methods were sequenced. First, the 72 kd region of a SDS-PAGE gel of HDL in which the 72 kd band was previously shown to be prominent was excised, then the protein extracted was digested by trypsin, analyzed by LC-MS/MS on LTQ-Orbitrap (Thermo), and identified by Discoverer software against the human part of the Uniprot database and a decoy database (in order to determine the false discovery rate). Second, HDL samples were analyzed by 2D gel electrophoresis. For the first dimension, Immobiline DryStrip NL (pH 3–7, 13 cm) (GE) was used. The strips were rehydrated in rehydration solution (40 mM DDT, 4% CHAPS, 8 M urea, 2 M thiourea, 2% IPG, and 1 *μ*l of 2% bromophenol blue). Isoelectric focusing was done with an Ettan IPGphor3 (GE) and maintained at 20°C. The program was 150 V for 4 h, 10 h gradient from 150 to 1000 V, 4 h gradient from 1000 to 2000 V, 2 h at 2000 V, 3 h gradient from 2000 to 3000 V, and finally 1 h at 3000 V (total, 26,850 Vh). The strips were equilibrated for 15 min in 0.05 M Tris-HCl (pH 8.8), 6 M urea, 30% (*v*/*v*) glycerol, 2% (*w*/*v*) SDS, and 20 mM DTT and then equilibrated for 15 min in the same buffer but containing 125 mM iodoacetamide in place of DTT. The equilibrated strips were applied to a vertical 8% SDS page used on S.E. 600 Ruby (Amersham Biosciences). The gel was silver stained in order to identify the protein spots which were excised and sent for sequencing.

### 2.6. Measurement of Vitamin E (*α*-Tocopherol) in HDL

HDL from 81 diabetic patients was purified by density gradient microultracentrifugation as described above. Aliquots (500 *μ*l) of HDL were mixed with 500 *μ*l of extractant and the samples shaken vigorously (vortexed for 10 min) with 6 ml of petroleum ether. Samples were then centrifuged at 800*g* for 5 min and the supernatants collected and divided into two equal aliquots. One aliquot was dried under nitrogen and stored at −70°C prior to analysis by HPLC. The other aliquot was dried under nitrogen, resuspended in 300 *μ*l of ethanol and the absorbance at 450 nm read by a VP Supersystem analyzer (Abbott). To measure *α*-tocopherol, the aliquot was resuspended in 50 *μ*l of ethanol and 25 *μ*l analyzed by HPLC. The column used was a Microsorb-MV C18 (5 *μ*m; 4.6 mm × 25 cm; Rainin) and the solvent was methanol flowing at 2 ml/min. The HPLC was a model 1090 (Hewlett-Packard) and the detector a UV diode array model 1040 (Hewlett-Packard). Components were monitored at 290 nm and the calculations were based on peak areas and the relative intensities determined using mixtures of authentic standards. The coefficient of variation between runs was 2.8% for *α*-tocopherol [12].

### 2.7. Reverse Cholesterol Transport (RCT) Assay

This assay, assessing the ability of serum from an individual to promote cholesterol efflux from macrophages, was described previously by Asleh et al. [9]. Briefly, J774 cells were seeded onto 24-well plates in DMEM medium with 8% FCS for 48 hours. Cells were incubated with ^3^H-labeled cholesterol (0.3 *μ*C/ml) in serum-free DMEM supplemented with 1% BSA in 37°C for 16 hours. After 2 washes with PBS, the cells were exposed to DMEM supplemented with oxidized HDL, with or without Hb/Hp complex samples for 3 hours. Medium was collected in scintillation tubes, and cells were incubated overnight with 0.1 M NaOH in 37°C. Radioactivity was measured both in cells and media to assess the RCT activity of serum.

### 2.8. Ethics Committee Approval to Use Human Samples

The use of human serum samples in this study was approved by the University of Pittsburgh Institutional Review Board (IRB, protocol number PRO09010072).

### 2.9. Statistical Analysis

Data are reported as the mean ± SD. In experiments comparing Hp 1-1 and Hp 2-2 only, the Student's *t*-test was used. In experiments comparing differences among the three Hp types, we used one-way ANOVA followed by post hoc comparisons between two specific Hp genotypes using Bonferroni's adjustment for multiple comparisons. A *p* value less than 0.05 was considered as statistically significant.

## 3. Results

### 3.1. Hb Cross-Linking with HDL Is Hp Genotype Dependent

**Figure 1:**
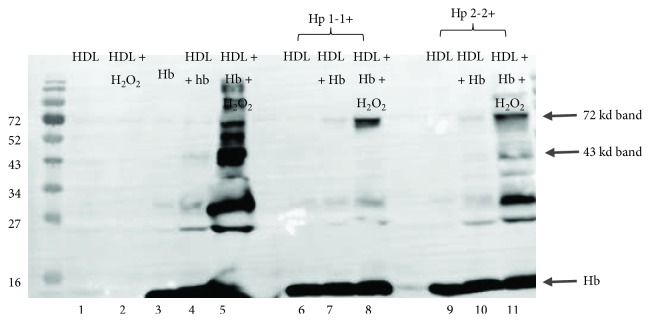
Detection of Hb cross-links on HDL by Western blot. Hp 1-1 or 2-2 was incubated with Hb (1 : 1 ratio) creating a Hp-Hb complex. These complexes, with or without H_2_O_2_ (1 : 10 molar excess), were added to HDL from healthy volunteers, loaded on a SDS-PAGE gel and were transferred to a PVDF membrane. (1) HDL, (2) HDL + H_2_O_2_, (3) Hb, (4) HDL + Hb, (5) HDL + Hb + H_2_O_2_, (6) HDL + Hp 1-1, (7) HDL + Hb/Hp 1-1, (8) HDL + Hb/Hp 1-1 + H_2_O_2_, (9) HDL + Hp 2-2, (10) HDL + Hb/Hp 2-2, and (11) HDL + Hb/Hp 2-2 + H_2_O_2_.

**Figure 2:**
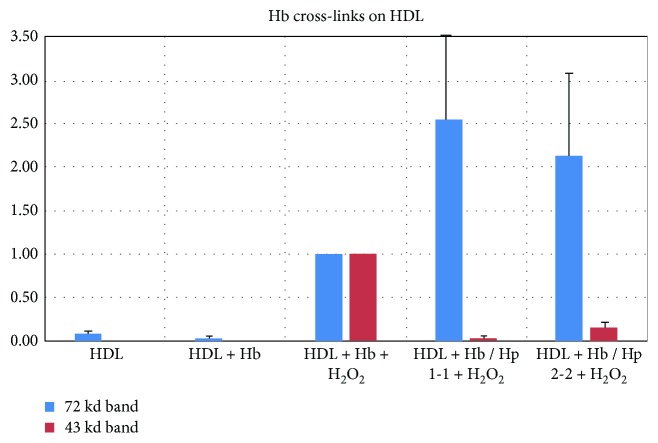
Measurement of Hb adducts with HDL. The 72 kd and 43 kd Hb immunoreactive bands were analyzed by ImageMaster, and the results were normalized to that obtained with HDL + Hb + H_2_O_2_ alone. The experiment was repeated eight times with 4 separate Hp preparations of each type (1-1 and 2-2) with each Hp preparation assessed twice. Data is reported as mean ± SD.

We assessed by Western blot the ability of Hp 1-1-Hb or Hp 2-2-Hb complexes to become cross-linked with HDL in the presence of H_2_O_2_ (Figure 1). In native HDL (lane 1), we found a weak Hb-immunoreactive 72 kd band that increased with the addition of Hb and H_2_O_2_, with or without Hp (lanes 5, 8, and 11). In the presence of Hb and H_2_O_2_, we observed additional bands (ranging from 25 kd to 52 kd) (lane 5) that indicated the formation of other Hb cross-linked species in HDL induced by Hb. The addition of Hp to this system reduced the intensities of several of these Hb HDL adducts, most notably a 43 kd species (average ± SD: 0.03 ± 0.04 (Hp 1-1), 0.16 ± 0.13 (Hp 2-2); median: 0.012 (Hp 1-1), 0.18 (Hp 2-2); post hoc pairwise comparison *p* value = 0.02, *n* = 8, comparing HDL + Hb + Hp 1-1 + H_2_O_2_ versus HDL + Hb + Hp 2-2 + H_2_O_2_). The differences in band intensities between HDL incubated with Hb, H_2_O_2_, and either Hp 1-1 or Hp 2-2 (lanes 8, 11) are summarized in Figure 2.

### 3.2. Hydroxyurea Prevents Hb Cross-Linking to HDL

**Figure 3:**
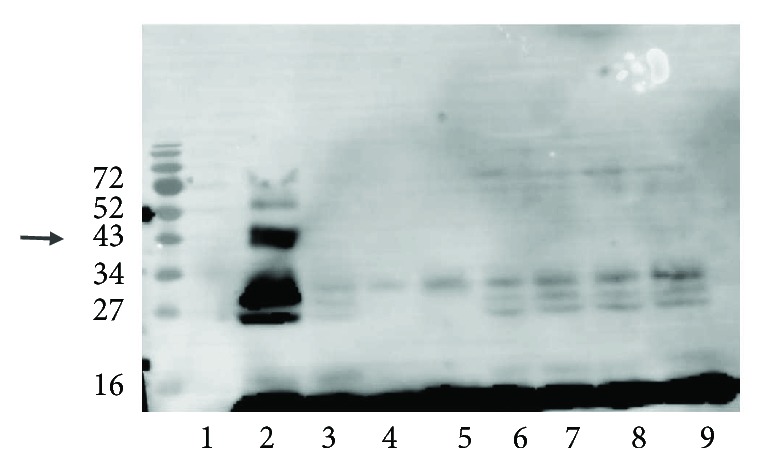
Hydroxyurea addition to HDL, Hb, and H_2_O_2_ inhibits Hb cross-link formation. HDL (obtained from the plasma of healthy volunteers), Hb, and H_2_O_2_ (1 : 10 ratio) were incubated with different concentrations of hydroxyurea for 1 hour. The samples were loaded on SDS-PAGE gel, transferred to a PVDF membrane, and stained with anti-Hb antibody. The sample order is (1) HDL, (2) HDL + Hb + H_2_O_2_, (3) HDL + Hb, (4) Hb, (5) Hb + H_2_O_2_, (6) HDL + Hb + H_2_O_2_ + hydroxyurea (400 nM), (7) HDL + Hb + H_2_O_2_ + hydroxyurea (200 nM), (8) HDL + Hb + H_2_O_2_ + hydroxyurea (100 nM), and (9) HDL + Hb + H_2_O_2_ + hydroxyurea (10 nM).

Hydroxyurea, a tyrosine radical blocker [13], was added to the Hb-H_2_O_2_-HDL incubation (Figure 3). The 43 kd band (arrow) is seen in HDL with Hb and H_2_O_2_ (lane 2), but it is inhibited by the addition of hydroxyurea (lanes 6–9).

### 3.3. HDL Reverse Cholesterol Transport Is Impaired When HDL Is Oxidized

**Figure 4:**
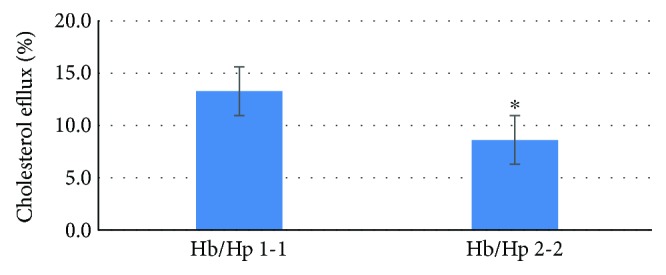
The effect of Hp genotypes on the cholesterol efflux of oxidized HDL. Hb-Hp1-1 or Hb-Hp 2-2 complexes were incubated with the same HDL preparation in the presence of H_2_O_2_ (1 : 10 molar excess) for 1 hour. After incubation, the samples were added to macrophages and the efflux of (^3^H) cholesterol from macrophages was measured (∗ indicates the *p* value < 0.001 (t-test) comparing Hb/Hp 1-1 versus Hb/Hp 2-2).

HDL was incubated with Hp 1-1-Hb or Hp 2-2-Hb complexes, and RCT stimulated by this HDL was measured (Figure 4). In the presence of Hb-Hp 1-1 complexes, the ability of HDL to promote cholesterol efflux was significantly higher than with HDL complexed with Hb-Hp 2-2 (average ± SD: 13.3 ± 3.8 and 8.6 ± 2.9 for Hp 1-1 and Hp 2-2, respectively; median 13.9 and 7.9 for Hp 1-1 and Hp 2-2, respectively, ^∗^*p* value < 0.001 (*t*-test) comparing Hb/Hp 1-1 versus Hb/Hp 2-2, *n* = 8).

### 3.4. Identification of the Proteins in the Hb Cross-Linked Species of Native HDL

**Figure 5:**
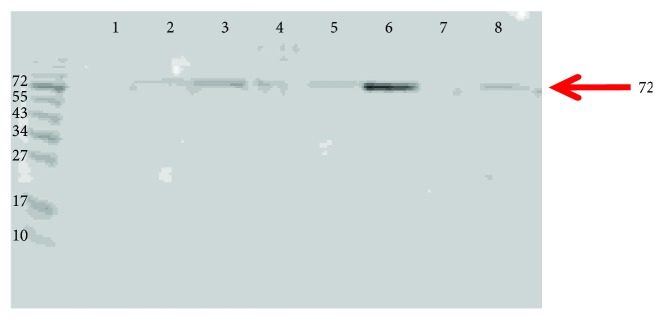
Detection of a 72 kd Hb immunoreactive band on native HDL. Eight HDL samples from diabetic individuals were loaded on an SDS-PAGE gel and transferred to a PVDF membrane. Anti-Hb antibody was added to the membrane, as described in Methods. A 72 kd band was seen (arrow) indicating the presence of a cross-linked species.

We investigated the presence of Hb cross-linked proteins on the HDL of individuals with different Hp genotypes. HDL was purified using a microultracentrifugation technique and subjected to PAGE. Western blot analysis using anti-Hb (see Figure 5) showed a 72 kd band (red arrow) which varied in intensity between individuals.

**Figure 6:**
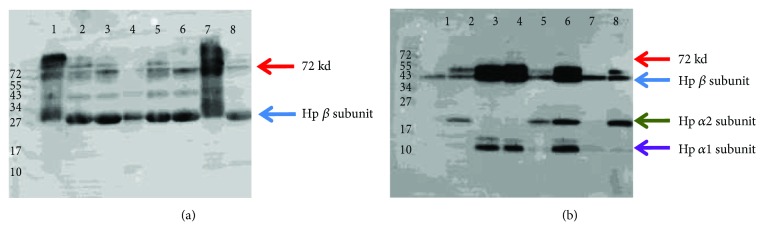
(a-b) Detection of cross-links on HDL with anti-ApoA1 and Hp antibodies. The same membrane from Figure 5 was reblotted and was incubated with anti-Apo A1 (panel a) or anti-Hp (panel b).

We hypothesized that the 72 kd species might contain Hb cross-linked to ApoA1 or Hp. We therefore performed Western blot analysis of the same membrane with anti-apoA1 and anti-Hp antibodies and found 72 kd anti-Apo A1 reactive bands but no 72 kd anti-Hp band (see Figure 6) suggesting that this 72 kd species contained Apo A1 but not Hp. Sequence analysis of the excised 72 kd band confirmed the presence of *α* and *β* subunits of Hb and Apo A1 as being part of this 72 kd complex.

### 3.5. Quantitation of the 72 kd Hb Immunoreactive Band in Hp 1-1 and Hp 2-2 DM Individuals

The amount of the 72 kd species was measured in 52 diabetic individuals by Western blot analysis. We found no significant difference in the amount of the 72 kd complex in individuals with the different Hp types (average ± SD: 0.12 ± 1.4 (Hp 1-1), 1.43 ± 2.7 (Hp 2-2); median: 0.07 (Hp 1-1), 0.3 (Hp 2-2), *n* = 17, *p* value = 0.19 (*t*-test) comparing Hp 1-1 versus Hp 2-2).

### 3.6. Quantification of Vitamin E Levels on HDL

The amount of vitamin E in the HDL from 73 DM individuals who participated in the HapE study was measured (blood collected at entry into the study), and the average vitamin E levels in HDL segregated by Hp type are seen in Table 1. We found a significant difference in HDL vitamin E levels between the three Hp genotypes (overall *p* value for difference between groups = 0.0165 (ANOVA)). There was a 43% lower vitamin E level in HDL from Hp 2-2 DM individuals as compared to Hp 1-1 DM individuals (post hoc pairwise comparison between Hp 1-1 versus Hp 2-2 *p* value = 0.0026 (*t*-test)). There was no significant difference in total serum vitamin E by Hp type (overall *p* value for difference between groups = 0.07 (ANOVA)) [14].

## 4. Discussion

We have shown here that Hb can form protein cross-links with HDL and that Hp 2-2 is inferior to Hp 1-1 in inhibiting the formation of these adducts with HDL. Previous work had established that there was more Hb associated with the HDL of Hp 2-2 DM individuals and that the HDL of these individuals is dysfunctional [5]. We have demonstrated here that the increased association of Hb and oxidative damage by Hb in Hp 2-2 can be recapitulated *in vitro* with purified Hp, Hb, and HDL. This suggests that it may be possible to use such an *in vitro* system to identify drugs that may similarly prevent HDL Hb adducts *in vivo*.

We have not definitively identified the nature of the protein adducts formed between Hb and HDL. MS sequencing of the 72 kd Hb immunoreactive band which appears in native HDL suggests that this complex includes apoA1. A 43 kd adduct of Hb with HDL and which is Hp type specific appears to also be an adduct of Apo A1 and Hb and this is consistent with the MW of these two proteins (27 kd and 16 kd). The Hp-binding site is on helix 6 of Apo-A1, and overlaps with the binding site of lecithin acetyl transferase (LCAT) on Apo-A1. This overlapping causes the displacement of LCAT and has been shown to result in an inhibition of LCAT cholesterol esterification rate *in vitro* and decreased LCAT activity significantly lower in Hp 2-2 diabetic individuals than in Hp 1-1 diabetic individuals [9]. This theory is consistent with the significant reduction in cholesterol efflux ability noted for HDL oxidized with Hp 2-2 (Figure 4). This hypothesis is further supported by Watanabe et al. who demonstrated that the Hp presence on HDL allows Hb to associate with HDL, and the amount of Hb in HDL increases HDL proinflammatory properties in CHD patients [14]. It will be of great interest to determine if the presence of the 72 kd Hb reactive species or the propensity of a given patient to form the 43 kd adduct is predictive of cardiovascular events and may therefore serve as a targetable biomarker.

We have also demonstrated for the first time that the concentration of vitamin E in HDL is Hp type dependent. In the patients in whom this was measured we had previously reported that total serum vitamin E was not Hp type dependent. The decreased amount of vitamin E in the HDL of Hp 2-2 individuals may be due to its increased consumption by the Hb which is bound to it. Similarly, Ji et al. have shown an association between the elevation of plasma Hb levels as a result of hemolysis in sickle cell anemia and decreased antioxidant function of HDL. This study extends the relevance of our findings to other diseases [15].

Supplementation of vitamin E would be expected to replenish vitamin E within HDL and thereby provide protection. These data may explain why vitamin E has been shown to improve HDL function in Hp 2-2 individuals but not in other Hp types and why vitamin E may be protective against CVD in Hp 2-2 but not in the other Hp types. It would be interesting to know if baseline HDL vitamin E levels were predictive of benefit from antioxidant therapy in recent antioxidant trials.

It is increasingly being understood that HDL function may be more important than HDL mass. Increasing HDL mass in individuals in whom HDL is dysfunctional may actually be harmful [7]. We have shown that the adducts formed here result in functional changes in the HDL. Ongoing studies are addressing whether these adducts can be used as surrogate markers for HDL functionality.

## Figures and Tables

**Table 1:** Measurement of vitamin E levels in HDL from diabetic individuals.

	Average ± SD vitamin E levels (*μ*g/mg HDL)	% change versus Hp 1-1
Hp 1-1	1.18 ± 0.62	—
Hp 2-1	0.99 ± 0.63	−17%
Hp 2-2	0.67 ± 0.62	−43%

Comparison between vitamin E levels in HDL from diabetic individuals with different Hp phenotypes. There was a significant difference in HDL vitamin E levels between the three Hp genotypes (overall *p* value for difference between groups = 0.0165 (ANOVA), post hoc comparison between Hp 1-1 and Hp 2-2 *p* value = 0.0026 (*t*-test)).

## Data Availability

All data arising from this study are contained within the manuscript.
